# Significant impact of circulating tumour DNA mutations on survival in metastatic breast cancer patients

**DOI:** 10.1038/s41598-021-86238-7

**Published:** 2021-03-24

**Authors:** Axel Muendlein, Kathrin Geiger, Stella Gaenger, Tobias Dechow, Christoph Nonnenbroich, Andreas Leiherer, Heinz Drexel, Andreas Gaumann, Wolfgang Jagla, Thomas Winder, Frank Mayer, Thomas Decker

**Affiliations:** 1 Molecular Biology Laboratory, Vorarlberg Institute for Vascular Investigation and Treatment, Stadtstraße 33, 6850 Dornbirn, Austria; 2 Medical Central Laboratories, 6800 Feldkirch, Austria; 3 Onkologie Ravensburg, 88212 Ravensburg, Germany; 4 Department of Internal Medicine, Hospital Bregenz, 6900 Bregenz, Austria; 5 Institute of Pathology Kaufbeuren-Ravensburg, 87600 Kaufbeuren, Germany; 6 Department of Haematology and Oncology, Academic Teaching Hospital Feldkirch, 6800 Feldkirch, Austria; 7 Praxis und Tagesklinik, Prof. Dr. Oettle Helmut und Prof. Dr. Dr. Mayer Frank, 88045 Friedrichshafen, Germany

**Keywords:** Breast cancer, Cancer genetics, Cancer therapy

## Abstract

Mutational analysis of circulating tumour (ct) DNA holds promise as an effective tool to predict the course of metastatic breast cancer (MBC). In the present study we used targeted next generation sequencing of ctDNA to evaluate the impact of cancer driven mutations on the prognosis of MBC. The study included 59 oestrogen receptor-positive (ER+), HER2-negative MBC patients. Sequencing analysis was performed in ESR1, PIK3CA, ERBB2, PTEN, TP53, KRAS, HRAS, NRAS, and AR. At baseline, patients started receiving either chemotherapy (34%; n = 20) or cyclin-dependent kinase 4/6 inhibitor therapy in combination with endocrine therapy (CDK4/6i+ET; 66%; n = 39). Overall, 64.4% (n = 38) of the patients carried at least one pathogenic or likely-pathogenic mutation. Number of ctDNA mutations was significantly linked with worse progression free survival (PFS; *p* = 0.003) and overall survival (OS; *p* = 0.007). Furthermore, ctDNA load, defined by the number of mutant ctDNA molecules per mL plasma, significantly correlated with PFS (*p* < 0.001) and OS (*p* = 0.001). Furthermore, mutational status of ESR1 and TP53 significantly predicted PFS (*p* = 0.024 and *p* = 0.035, respectively) and OS (*p* < 0.001 and *p* = 0.035, respectively). These results emphasizes the clinical value of ctDNA mutational analysis in the management of advanced breast cancer.

## Introduction

Breast cancer is the most common cancer in women worldwide affecting eight to ten women during lifetime. Despite a substantially progress in therapy, metastatic breast cancer is still considered incurable. Therapy goals are prolongation of survival and maintaining quality of life^1^. Cyclin-dependent kinase 4/6 inhibitors in combination with targeted endocrine therapy (CDK4/6i+ET), have been recommended as standard therapy for the treatment of oestrogen receptor positive (ER+), HER2 negative metastatic breast cancer (MBC)^2^. In addition, chemotherapy is still frequently used, in particular in patients with endocrine resistance or in patients with immediately life-threatening disease and the need for rapid treatment response^3, 4^.

Patients' responses to both targeted therapies and chemotherapies are often quite variable and limited by de novo and acquired resistance leading to disease recurrence^5^. Therefore, tools are required to predict treatment response, as well as to identify resistance to select patients who will likely benefit from therapy. Novel technologies including next generation sequencing (NGS) and digital PCR allow the analysis of cell-free DNA (cfDNA) from plasma samples, known as liquid biopsies, linking levels of circulating tumour DNA (ctDNA) or specific tumour-derived mutations to prognosis and therapy response in cancer patients, including those with ER+ , HER2 negative breast cancer.

In this regard, the BOLERO-2 trial^6^ and other studies^7–9^ identified variants in the ESR1 gene as predictive markers in advanced breast cancer patients under aromatase inhibitor therapy. The PALOMA-3 study showed the genesis of new driver mutations in PIK3CA and ESR1 in patients receiving the selective oestrogen receptor degrader fulvestrant, alone or in combination with CDK4/6i^10^. Furthermore, Kruger et al. showed that a low ctDNA load correlated with a prolonged progression free survival (PFS) in MBC patients treated with everolimus and exemestane^11^. Therefore, mutational analysis of ctDNA holds promise as an effective tool to predict the course of MBC. However, ctDNA profiling is still challenging, in particular due to the limited amount and high fragmentation of cfDNA, the low frequency of ctDNA mutations, and the susceptibility to errors of NGS-based techniques. Hence, number of studies investigating the impact of ctDNA on the prognosis of ER+/HER2 negative MBC is still limited and no provisional guideline recommendations for ctDNA testing in liquid samples of MBC patients exist, so far. Therefore, in the present study we performed targeted deep sequencing of ctDNA from a panel of nine genes (ESR1, PIK3CA, ERBB2, PTEN, TP53, KRAS, HRAS, NRAS, and AR), previously associated with therapy resistance, and the course of MBC^10–16^ to further evaluate the clinical validity of ctDNA analysis in a consecutively recruited population of ER+/HER2 negative MBC patients.

## Results

### Patients’ characteristics

During recruitment period, 71 patients gave written informed consent for participation in the present study. Out of these, four patients did not meet the inclusion criteria and three patients were lost to follow up. DNA extraction failed in five patients. Consequently, the present study included 59 patients. Median age of our patients was 66.2 (55.6–76.1) years. At baseline, 39 patients (66.1%) started therapy with CDK4/6i (ribociclib, palbociclib or abemaciclib) combined either with an aromatase inhibitor (letrozole or exemestane; n = 33) or with a selective oestrogen receptor degrader (fulvestrant; n = 6). Out of these patients, 33 subjects started first line-therapy and 6 s line-therapy. Further 20 patients (33.9%) started chemotherapy (paclitaxel plus bevacizumab, n = 9; capecitabine plus bevacizumab; n = 10; carboplatin and gemcitabine, n = 1). Thirteen patients assigned to chemotherapy started first line-therapy and 7 s line-therapy. Therefore, at baseline, 46 (78%) patients started first and 13 (22%) patients started second line therapy. Clinicopathological characteristics stratified by the scheduled therapy (CDK4/6i+ET versus chemotherapy) are given in Table 1.

**Table 1:** Basic clinical and clinicopathological characteristics stratified by scheduled therapy.

	CDK4/6 inhibitor therapy with endocrine therapy (N = 39)	Chemotherapy (N = 20)	*p* value
**Median age, years (IQR)**	67.2 (60.1–77.0)	62.2 (54.1–73.3)	0.209
**Menopausal status**			
Pre/peri	8 (20.5)	5 (25.0)	0.694
Post	31 (79.6)	15 (75.0)	
**Histology of primary tumour**			
NST, N (%)	29 (74.4)	15 (75.0)	0.957
ILC, N (%)	10 (25.6)	5 (25.0)	
**Metastatic sites**			
Visceral, N (%)	22 (56.4)	15 (75.0)	0.162
Non-visceral, N (%)	17 (43.6)	5 (25.0)	
**Number of metastatic sites**			
1	22 (56.4)	4 (20.0)	
2	13 (33.3)	8 (40.0)	0.007
≥ 3	4 (10.3)	8 (40.0)	
**Prior adjuvant endocrine therapy**			
No, N (%)	23 (59.0)	4 (14.8)	0.004
Yes, N (%)	16 (41.0)	16 (80.0)	
**Prior 1st line therapy (metastatic setting)**			
Chemotherapy only, N (%)	2 (33.3)	0 (0.0)	0.097
Endocrine therapy, N (%)	4 (66.7)	7 (100.0)	
**Scheduled therapy line**			
1st, N (%)	33 (84.6)	13 (65.0)	0.088
2nd, N (%)	6 (15.4)	7 (35.0)	

Abbreviations: IQR, interquartile range; NST, Invasive carcinoma of no special type; ILC, invasive lobular carcinoma.

### Circulating tumour DNA mutations

The mean sequencing read coverage per sample was 34,460x. In total, 49 different putative tumour-derived DNA variants across 7 genes were identified after filtering for germline variants and exclusion of low-confidence variants applying the variant caller algorithm smCounter2, integrated in the used web-based data analysis tool GeneGlobe (Qiagen, Hilden, Germany). According to smCounter2 variant caller, three variants (NRAS p.Ser117Asn, TP53 p.Tyr377Pro, and AR p.Gln59Leu) did not pass all applied quality filters and were excluded from further analysis. One variant, TP53 p.Arg213Arg, was found in four patients with variant allele frequencies around 50% suggesting to be germline rather than somatic and, therefore, was also excluded. Out of the remaining 45 variants, 36 variants have been previously reported in the COSMIC v92 database or in the NCBI dbSNP database providing data on pathogenicity for 29 variants (27 variants have been classified as pathogenic and two variants as benign). Using in silico bioinformatic tools SIFT^17^, PolyPhen-2^18^ or ‘Splice Site Prediction by Neural Network’^19^ for classification of the yet 16 unclassified variants, 14 variants could be classified as likely-pathogenic or pathogenic and two variants as neutral. Characteristics of all determined true variants including variant allele frequencies and number of mutant ctDNA molecules per mL plasma for each patient are given in Supplementary Table S1.

Overall, 64.4% (n = 38) of the patients carried at least one pathogenic or likely-pathogenic mutation and 10.2% (n = 6) subjects carried at least three different putative causal mutations. The most frequently mutated gene was PIK3CA (28.8%; n = 17), followed by TP53 (18.6%; n = 11), ESR1 (16.9%; n = 10), PTEN (13.6%; n = 8), AR (8.5%; n = 5), and ERBB2 (3.4%; n = 2). No true mutations were found in KRAS, NRAS, and HRAS. The most prevalent individual mutation was PIK3CA p.His1047Arg (10.2%; n = 6), followed by PIK3CA p.Glu545Lys (6.8%; n = 4), ESR1 p.Asp538Gly (6.8%; n = 4), ESR1 p.Glu380Gln (5.1%; n = 3), and AR p.Gln58Leu (5.1%; n = 3). Prevalence of ESR1 mutations was significantly associated with endocrine therapy prior baseline examination (OR = 11.50 [1.30–94.00]; *p* = 0.009), particularly occurring in those patients with prior aromatase inhibitor (37.5%) or fulvestrant (100%) therapy (Supplementary Fig. S1).

### Impact of ctDNA on the course of MBC

**Figure 1:**
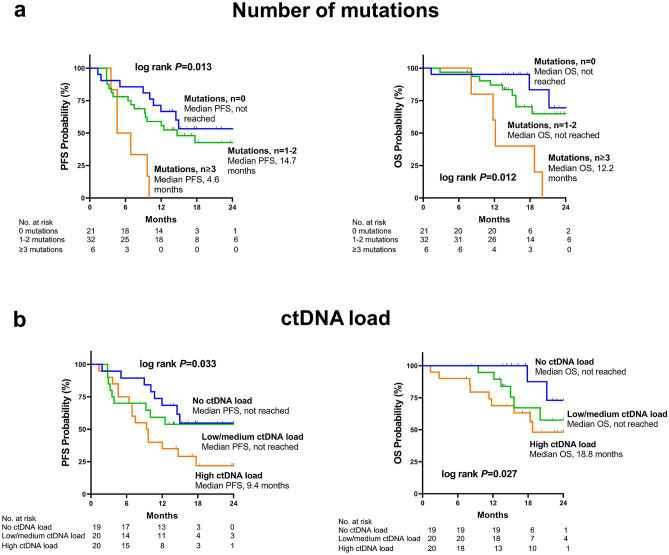
Kaplan–Meier estimates of survival according to (**a**) mutation number (**a**) and (**b**) ctDNA load. Number of likely-pathogenic or pathogenic mutations was categorized as 0 mutations, 1–2 mutations, and ≥ 3 mutations. Number of mutant ctDNA molecules per mL plasma was used to assess ctDNA load, which was categorized into tertiles. No (zero) ctDNA load was defined by the first tertile, a low/medium ctDNA load was defined by the second tertile, and a high ctDNA load was defined by the third tertile of mutant ctDNA molecule concentration. P-values were obtained by Log-Rank-Mantel-Cox-tests. PFS, progression-free survival; OS, overall survival.

During a median follow-up period of 18.0 (95% CI:15.2–22.1) months, 54.2% of the patients (n = 32) showed a progression of the disease and 28.8% of the patients (n = 17) died. The number of pathogenic or likely-pathogenic mutations was significantly linked with worse PFS (HR _per mutation_ = 1.51 [1.14–1.98]; *p* = 0.003) and OS (HR _per mutation_ = 1.51 [1.12–2.05]; *p* = 0.007). Kaplan–Meier survival curves together with results from Log-Rank-Mantel-Cox-tests according to zero mutations, 1–2 mutations, and three or more mutations are shown in Fig. 1a.

Furthermore, ctDNA load, defined by the number of mutant ctDNA molecules per mL plasma, significantly correlated with PFS (HR _per SD_ = 2.00 [1.36–2.96]; *p* < 0.001) and OS (HR _per SD_ = 1.93 [1.31–2.84]; *p* = 0.001). Patients were categorized according to tertiles of the number of mutant ctDNA molecules per mL plasma for Kaplan–Meier survival analysis (Fig. 1b). PFS and OS significantly decreased from patients without any mutation (1st tertile; ctDNA load = 0 mutant ctDNA molecules per mL plasma) over those with a low or medium ctDNA load (2nd tertile; median ctDNA mutant molecules per mL plasma = 74.2; IQR: 29.8–133.2) to those with the highest ctDNA load (3rd tertile; median mutant ctDNA molecules per mL plasma = 879.2; IQR: 384.4–2087.2).

**Figure 2:**
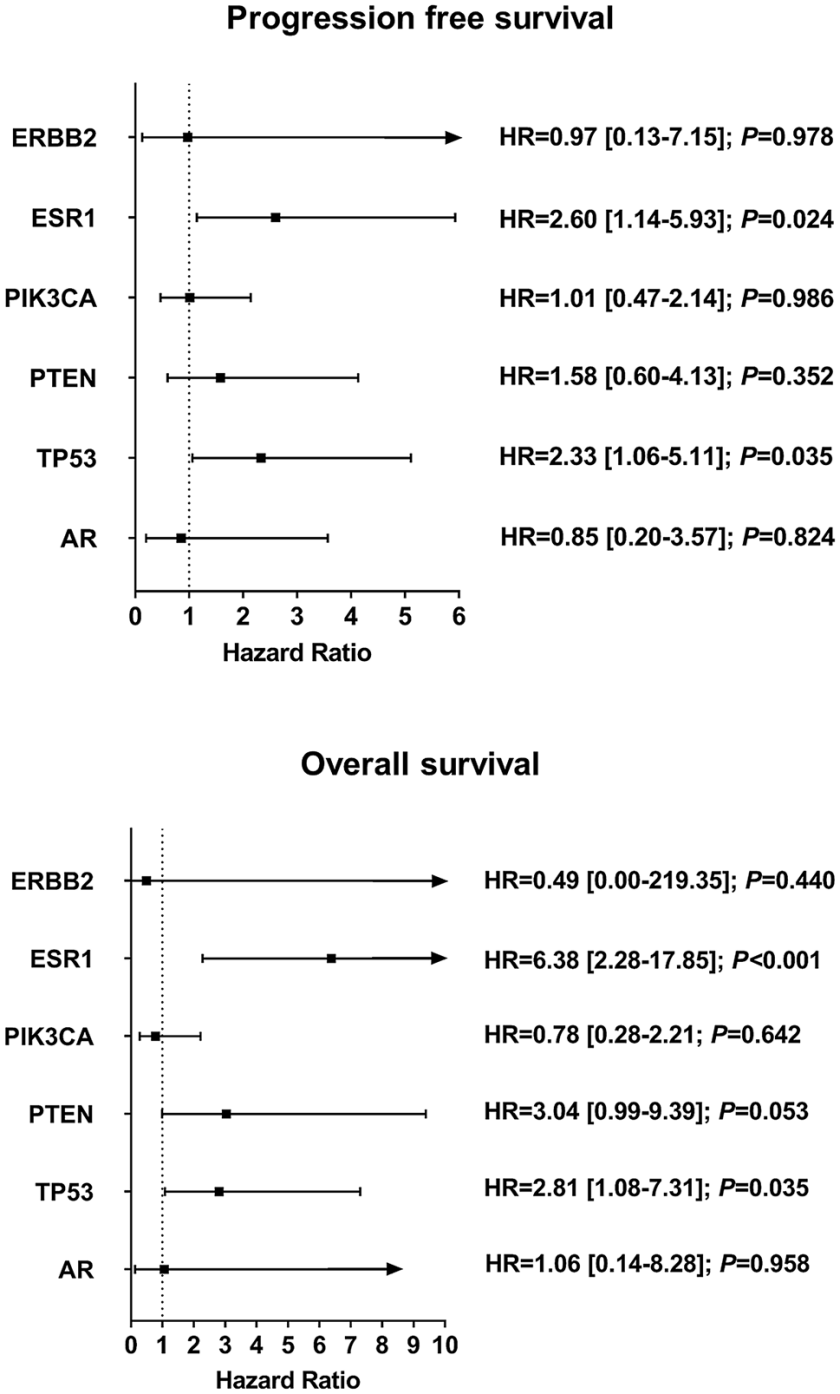
Hazard ratios [95% confidence interval] of mutated genes for progression free survival and overall survival.

Impact of individual mutated genes as well as of likely-pathogenic or pathogenic hot-spot mutations (affecting more than one patient) on the course of MBC is shown in Fig. 2 and Supplementary Fig. S2, respectively. ESR1 p.Tyr537Cys and ESR1 p.Asp538Gly were significantly associated with PFS; ESR1 p.Tyr537Ser, ESR1 p.Tyr537Cys, ESR1 p.Asp538Gly, and PIK3CA p.Glu453Lys were significantly associated with OS. Patients with a likely-pathogenic or pathogenic mutation in ESR1 or TP53 were at increased risk for disease progression and death. Occurrence of mutated PIK3CA, ERBB2, PTEN or AR did not significantly influence PFS or OS.

**Figure 3:**
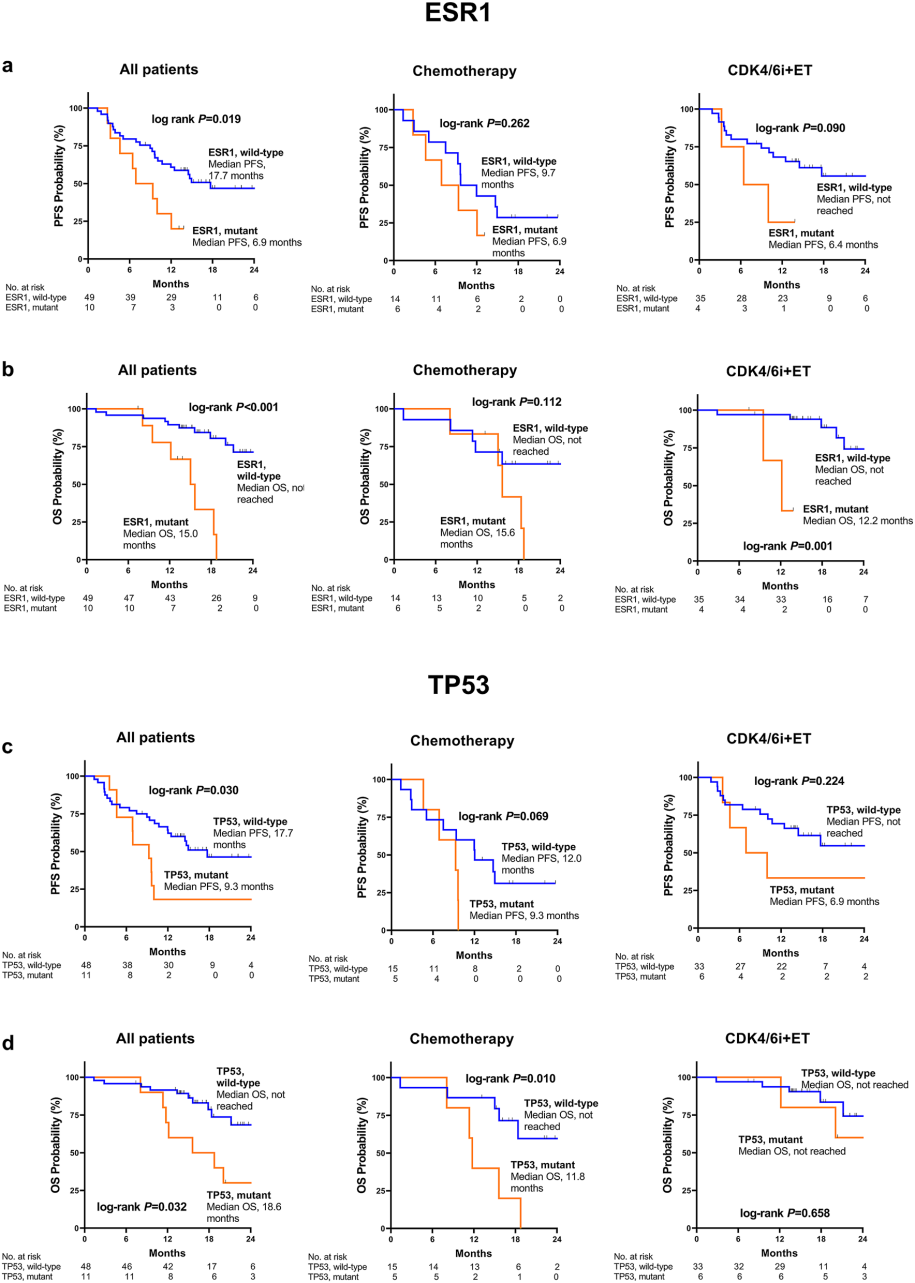
Kaplan–Meier estimates of survival according to (**a**, **b**) mutant ESR1 and (**c**, **d**) mutant TP53. P-values were obtained by Log-Rank-Mantel-Cox-tests. PFS, progression-free survival; OS, overall survival; CDK4/6i+ET, cyclin-dependent kinase 4/6 inhibitors in combination with endocrine therapy.

Furthermore, subgroup analysis based on the subsequent treatment (CDK4/6i+ET versus chemotherapy) after baseline sample collection was performed. Mutant ESR1 was significantly associated with OS in patients starting CDK4/6i+ET (HR = 15.05 [2.00–113.46]; *p* = 0.009), but not in patients starting chemotherapy (HR = 2.65 [0.76–9.22]; *p* = 0.126). Conversely, the impact of mutant TP53 on OS was stronger in patients starting chemotherapy (HR = 4.53 [1.29–15.98]; *p* = 0.019), compared to patients starting CDK4/6i+ET (HR = 1.50 [0.27–7.74]; *p* = 0.659). Regarding PFS, in subgroup analysis with respect to subsequent CDK4/6i+ET or chemotherapy the association between mutated ESR1 (HR = 2.87 [0.80–10.30]; *p* = 0.105 and HR = 1.88 [0.61–5.79]; *p* = 0.269, respectively) or mutated TP53 (HR = 1.99 [0.64–6.15]; *p* = 0.233 and HR = 2.91 [0.87–9.71]; *p* = 0.082, respectively) and survival did not reach statistical significance. Respective Kaplan–Meier survival curves together with results from Log-Rank-Mantel-Cox-tests for ESR1 and TP53 are shown in Fig. 3a–d.

## Discussion

In the present study we demonstrate that ctDNA mutational status of ESR1 and TP53 as well as number of ctDNA mutations and ctDNA load significantly predict outcome in oestrogen receptor-positive, HER2-negative metastatic breast cancer patients. Subgroup analysis with respect to therapy (CDK4/6i+ET versus chemotherapy) showed, that patients with mutant ESR1 and CDK4/6i+ET exhibit a reduced overall survival, while the association between mutant ESR1 and survival was not significant in patients treated with chemotherapy.

The crucial role of mutant ESR1 as a driver of resistance and worse outcome in MBC patients treated with aromatase inhibitors has been previously demonstrated by several clinical studies^6–9, 20^. In vitro studies have shown that mutations within the ESR1 ligand-binding-domain exhibit constitutive transcriptional activity of oestrogen receptor target genes conferring to full resistance under oestrogen-deprived conditions mimicking aromatase inhibitor treatment^21^. In vitro studies also suggest that ESR1 ligand-binding-domain mutations lead to a partial resistance to fulvestrant probably caused by a conformational change of the oestrogen receptor reducing its affinity to fulvestrant^21–23^. Notably, MBC patients with ESR1 mutations receiving a fulvestrant-containing therapy had a better prognosis compared to patients on aromatase inhibitors^20, 24^ pointing to the lower impact of ESR1 mutations on fulvestrant resistance compared to aromatase inhibitor resistance.

It should be stressed, however, that CDK4/6i are now standard of care^2^ and no clinical study compared the impact of ESR1 mutations on treatment response of CDK4/6i plus aromatase inhibitor therapy versus CDK4/6i plus fulvestrant therapy, so far. Limited patient sample size of our study hinders respective subgroup analysis. That said, we observed a significant accumulation of ESR1 mutations in patients treated with fulvestrant prior to baseline suggesting a particular role of ESR1 mutations in conferring fulvestrant resistance. This finding is in line with other studies^10, 25^ including the PALOMA-3 trial^10^ reporting a positive selection of ESR1 mutations in patients treated with fulvestrant, alone or in combination with CDK4/6i, although no significant impact of ESR1 mutations on PFS could be observed^9^. However, data regarding OS are pending, so far. Our study indicates that CDK4/6 inhibition in combination with either aromatase inhibitors or fulvestrant does not prevent from endocrine therapy failure triggered by ESR1 mutations. Further studies are needed to elucidate the impact of ESR1 mutations on endocrine resistance in patients receiving CDK4/6i+ET.

Mutant TP53 was significantly associated with PFS and OS in our study. This observation is well in concordance with other studies indicating TP53 mutational status as a biomarker of poor prognosis in breast cancer patients^26–29^. Subgroup analysis based on given therapy suggests that the impact of mutant TP53 on overall survival is stronger in patients receiving chemotherapy based regimes versus patients under CDK4/6i+ET. Previous reports of the association between TP53 mutation status and response to therapy have been inconsistent and results depend mostly on pre-defined clinical outcomes and treatments given^30^. Most of our patients treated with chemotherapy received either paclitaxel or capecitabine, an oral prodrug of 5-fluorouracil (5-FU), in combination with bevacizumab. These findings are confirmed by several other studies showing that breast cancer patients with TP53 mutant tumours had a worse outcome under 5-FU or taxane based chemotherapy compared to TP53 wild-type patients^31–34^.

Regarding CDK4/6i+ET, recently updated analyses of the MONALEESA-2 study as well as of the PALOMA-3 study showed that mutant TP53 was associated with poor survival in patients receiving either CDK4/6i+ET or placebo plus ET^35, 36^. Also in our study patients treated with CDK4/6i+ET and with TP53 mutations showed a shorter survival than those with wild-type TP53, but the association between mutant TP53 and survival did not reach statistical significance in this subgroup. Differences in the strength of association between TP53 mutation status and survival with respect to given therapy as observed in our study warrants further investigation.

Increased number of pathogenic mutations as well as elevated levels of ctDNA were significantly associated with worse outcome in our study. The prevalence of ctDNA mutations has been associated with the number of treatment lines^7, 14^ pointing to the presence of already existing treatment-resistant clones prior scheduled therapy resulting in shorter survival. On the other hand, elevated ctDNA levels have been linked with increased tumour size, tumour burden and tumour progression^37^. Several studies used allele frequencies of determined somatic mutations to quantify ctDNA levels^14, 38, 39^. However, van Dessel et al. showed that variant allele frequencies may be biased by preanalytical conditions, in particular by lysis of leucocytes causing higher numbers of wild-type alleles, while the number of mutant molecules per mL plasma remains mostly stable^40^. Therefore, we and others^11, 41^ used the number of mutant ctDNA molecules per mL plasma to assess ctDNA load.

It should be noted, that somatic alterations may also be present in cfDNA of aged individuals without cancer due to the clonal outgrowth of haematopoietic cells with acquired mutations, a process known as clonal haematopoiesis^42^. The most commonly detected mutations are from genes, which are frequently mutated in haematological malignancies such as DNMT3A, TET2, and ASXL1, and include also TP53. However, the prevalence of mutant TP53 due to clonal haematopoiesis in subjects without cancer is well below 1%^42^ indicating that the TP53 mutations detected in our study are rather derived from metastatic cancer than from clonal haematopoiesis.

Our study has strengths and limitations. By design, our study population was composed of consecutively recruited ER+/HER2 negative MBC patients treated either with chemotherapy or CDK4/6i+ET; our results, therefore, are not necessarily applicable to other breast cancer subtypes, breast cancer stages or treatment regimens. However, the most common subtype of MBC is ER+/HER2 negative MBC. Moreover, CDK4/6i+ET now represent standard therapy for the treatment of ER+/HER2 negative MBC, while chemotherapy is still commonly used, even in the absence of visceral crisis^2–4, 43^. Therefore, our study population, although not uniformly treated, covers a large proportion of MBC patients seen in clinical practise providing real-world data linking impact of cfDNA mutations with the course of MBC. Furthermore, we cannot exclude that some associations not found to be significant in our study may have reached statistical significance with a larger population or a longer follow-up period. That said, results from our study showing significant associations of mutant ESR1, mutant TP53, ctDNA load, and number of ctDNA mutations with worse outcome are well in line with the current literature confirming previous reports also in a moderate study population. In addition, our study suggests that strength of association between TP53 mutation status and outcome is stronger in patients on chemotherapy compared to patients on CDK4/6i+ET. However, limited sample size does not allow a more detailed subgroup analysis based on individual therapy regimes. Therefore, some aspects such as to compare the impact of mutant ESR1 on treatment response of CDK4/6i in combination with different ETs could not been taken into account and have to been further clarified in future studies. Our targeted NGS approach was designed to assess the impact of putative predictive or prognostic ctDNA markers on the course of MBC, but includes only a limited number of candidate genes and hot-spot regions. In this regard, our panel did not target RB1 mutations, which have been initially linked to CDK4/6i resistance^44^. However, RB1 mutations show a relatively low prevalence suggesting that mutant RB1 is not a major mechanism of CDK4/6i resistance^10^. Therefore, we decided not to include RB1 into our NGS gene panel. Similarly, genes found commonly mutated in MBC but without suspected actionable mutations, such as GATA3 or KMT2C^12, 13^, were not included in NGS analysis. However, the manageable number of selected regions of interest used in our approach allows high sequencing read coverage, necessary for somatic mutation detection in cfDNA, also when using low or medium throughput NGS instruments, such as the widely used Illumina MiSeq sequencer.

In conclusion, we observed significant associations of mutant ESR1, mutant TP53, ctDNA load, and the number of ctDNA mutations, respectively, with worse outcome in ER+/HER2 negative MBC patients. In addition, our study suggests that strength of association between TP53 mutation status and outcome is stronger in patients on chemotherapy compared to patients on CDK4/6i+ET. Our results are well in line with previous reports and confirm the high reliability of a targeted PCR-based NGS approach using unique molecular identifiers for error correction, emphasizing the clinical value of ctDNA mutational analysis in the management of advanced breast cancer.

## Materials and methods

### Study subjects

From October 2017 through June 2019, MBC patients were assessed for eligibility at the ‘Oncology Study Center Ravensburg’ (Ravensburg, Germany). Eligible patients were aged ≥ 18 years diagnosed with ER+/HER2 negative MBC starting first line or second line therapy with either CDK4/6i+ET or chemotherapy. MBC patients with other scheduled therapies were not included. All patients had to give written informed consent for participation in the present study. Patients were followed up until disease progression, death, or end of the observational period at June 12th, 2020. The primary endpoints were PFS (defined as objective disease progression or death) and overall survival (OS). The study protocol was approved by the Ethics Commission of the State Chamber of Medicine of Baden-Württemberg (Germany) and by the Ethic Committee of the State of Vorarlberg (Austria) and is in accordance with the 1964 Helsinki declaration and its later amendments or comparable ethical standards.

### Blood sampling and DNA extraction

At baseline, venous blood was collected into three 10 mL Cell-Free DNA BCT tubes (Streck Corporate, La Vista, NE). Cell-Free DNA BCT tubes contain a preservative which stabilizes nucleated blood cells for up to 7 days total minimizing cfDNA contamination from genomic DNA^45, 46^. cfDNA extraction and sequencing analysis was performed at the ‘Vorarlberg Institute for Vascular Investigation and Treatment’ (Dornbirn, Austria). Blood collection tubes were centrifuged for 10 min at room temperature at 1,600 × g. The supernatant of the plasma fractions were transferred into 2 mL microcentrifuge tubes followed by a second centrifugation step at 6,000 × g for 10 min to remove remaining cell debris and fragments. The supernatant of the second plasma fractions was immediately stored at − 80 °C until cfDNA extraction. CfDNA was isolated from 4 mL thawed plasma with the QIAmp Circulating Nucleic Acid Kit (Qiagen) according to the manufacturer’s instructions and finally eluted with 30µL elution buffer into 1.5 mL DNA low binding tubes (VWR International, Vienna, Austria). Extracted cfDNA was quantified using the Qubit dsDNA HS Assay together with the Qubit 3.0 Fluorometer (Thermo Fisher Scientific, Waltham, MA).

### Targeted next generation sequencing analysis

We selected 41 genomic regions across nine candidate genes (all coding exons of TP53 and PTEN and mutation hotspots in ESR1, PIK3CA, ERBB2, KRAS, HRAS, NRAS, and AR), for targeted deep sequencing analysis. Chromosomal positions of the selected regions of interest are given in Supplementary Table S2.

The QIAseq Targeted DNA Custom Panel Kit (Qiagen) was used for library construction. Primer design for library construction was performed by Qiagen. Because cfDNA is generally characterized by minor quality and high fragmentation compared to leukocyte genomic DNA, a dual stranded strategy with high density primer tiling was used generating 207 amplicons to fully cover the selected regions of interest at sequencing lengths of at least 100 bp.

Libraries were prepared following the protocol for Illumina instruments given in the QIAseq Targeted DNA Panel Handbook. We used the highest possible DNA input volume (15.5 µL) to maximize DNA input amounts. DNA samples were first fragmented, end-repaired and A-tailed in a single reaction. The prepared DNA fragments were then ligated at their 5′ ends to a specific adapter containing a unique molecular index (UMI) and a first sample-specific index. The use of UMIs allows for true low-frequency variant calling by mostly eliminating library amplification and sequencing artefacts^47, 48^. For target enrichment, ligated DNA molecules underwent targeted PCR using one region specific primer and one universal primer complementary to the adapter. Finally, a universal PCR was carried out to amplify the library and to integrate specific adapter sequences for Illumina instruments and additional sample-specific indices.

Libraries were quantified by quantitative real time PCR using the QIAseq Library Quant Assay Kit (Qiagen) together with a LightCycler 480 Real-Time PCR System (Roche Diagnostics, Vienna, Austria). Fragment size of the libraries were checked with the DNA High Sensitivity Kit on a 2100 Bioanalyzer instrument (Agilent, Santa Clara, CA).

Libraries were diluted to a concentration of 4 nM and between 7 and 10 libraries with different sample indexes were combined for subsequent sequencing analysis using a final library concentration of 15 pM. Sequencing of pooled libraries was performed on a MiSeqDx sequencer (Illumina, San Diego, CA) using the MiSeq Reagent Kit v2 (300-cycles) according to the manufacturer's instructions.

### Sequencing data analysis

FASTQ files were upload into the GeneGlobe data analysis center (Qiagen) for primary data analysis. This web-based tool filters, maps and aligns reads, as well as counts unique molecular barcodes associated with amplified genomic regions, and calls variants with a barcode-aware algorithm, called smCounter2^49^. Identified candidate variants were manually verified in the Integrative Genomics Viewer^50^. Verified sequence variants were checked for previously published reports in the ‘Catalogue of Somatic Mutations In Cancer’ (COSMIC) v92^51^ and in the NCBI dbSNP database^52^. Effect of previously undescribed missense mutations was evaluated with in silico bioinformatic tools ‘sorting intolerant from tolerant’ (SIFT; http://sift.jcvi.org)^17^ and ‘polymorphism phenotyping v2’ (PolyPhen-2; http://genetics.bwh.harvard.edu/pph2)^18^. Effect of potential splice site variations was evaluated by the ‘Splice Site Prediction by Neural Network’ (http://www.fruitfly.org/seq_tools/splice.html)^19^ tool.

The number of mutant ctDNA molecules per mL plasma was used to assess ctDNA load, as suggest by Kruger et al.^11^. In patients affected by more than one mutation, the highest mutant molecule concentration was used for statistical analysis.

### Statistics

Differences in baseline characteristics were tested for statistical significance with the Chi-squared tests for categorical and with Student’s t-test for continuous variables, respectively. Continuous variables are presented as medians and interquartile ranges (IQR). Survival times were calculated from date of inclusion until progression or death. Survival curves were generated using the Kaplan–Meier method and compared using the Log-Rank-Mantel-Cox-tests. Hazard ratios (HRs) and 95% confidence intervals of the HRs were derived from Cox proportional hazards models. Continuous variables were z-transformed before entered into regression analysis, providing HRs [95% confidence interval] per standard deviation (SD). Statistical analyses were performed with SPSS 25.0 (IBM, Armonk, NY) and GraphPad Prism 8 (GraphPad Software, San Diego, CA).

## Data availability

The data that support the findings of this study are available are available from the corresponding author on reasonable request.

## Supplementary Information

Supplementary Information

Supplementary Table S1
