# Supplementary material for: Significant impact of circulating tumour DNA mutations on survival in metastatic breast cancer patients
Source: Sci Rep. 2021 Mar 24;11:6761. doi: 10.1038/s41598-021-86238-7 (PMC7990915; doi:10.1038/s41598-021-86238-7)
Supplement: Supplementary file 1 — Supplementary Information [file 41598_2021_86238_MOESM1_ESM.pdf]

SUPPLEMENTARY INFORMATION

Significant impact of circulating tumour DNA mutations on survival in metastatic breast cancer patients

Axel Muendlein[1*], Kathrin Geiger[1,2], Stella Gaenger[1], Tobias Dechow[3], Christoph Nonnenbroich[3], Andreas Leiherer[1,2], Heinz Drexel[1,4], Andreas Gaumann[5], Wolfgang Jagla[5], Thomas Winder[6], Frank Mayer[7], Thomas Decker[3]

[1] Vorarlberg Institute for Vascular Investigation and Treatment, Molecular Biology Laboratory, 6850 Dornbirn, Austria; [2] Medical Central Laboratories, 6800 Feldkirch, Austria [3] Onkologie Ravensburg, 88212 Ravensburg, Germany; [4] Department of Internal Medicine, Hospital Bregenz, 6900 Bregenz, Austria; [5] Institute of Pathology Kaufbeuren-Ravensburg, 87600 Kaufbeuren, Germany; [6] Department of Haematology and Oncology, Academic Teaching Hospital Feldkirch, 6800 Feldkirch, Austria; [7] Praxis und Tagesklinik Prof. Dr. Oettle Helmut Prof. Mayer Frank, 88045 Friedrichshafen, Germany

*Corresponding author: Dr. Axel Muendlein; Vorarlberg Institute for Vascular Investigation and Treatment, Molekularbiologisches Labor, Stadtstraße 33; 6850 Dornbirn, Austria. E-Mail: axel.muendlein@vivit.at

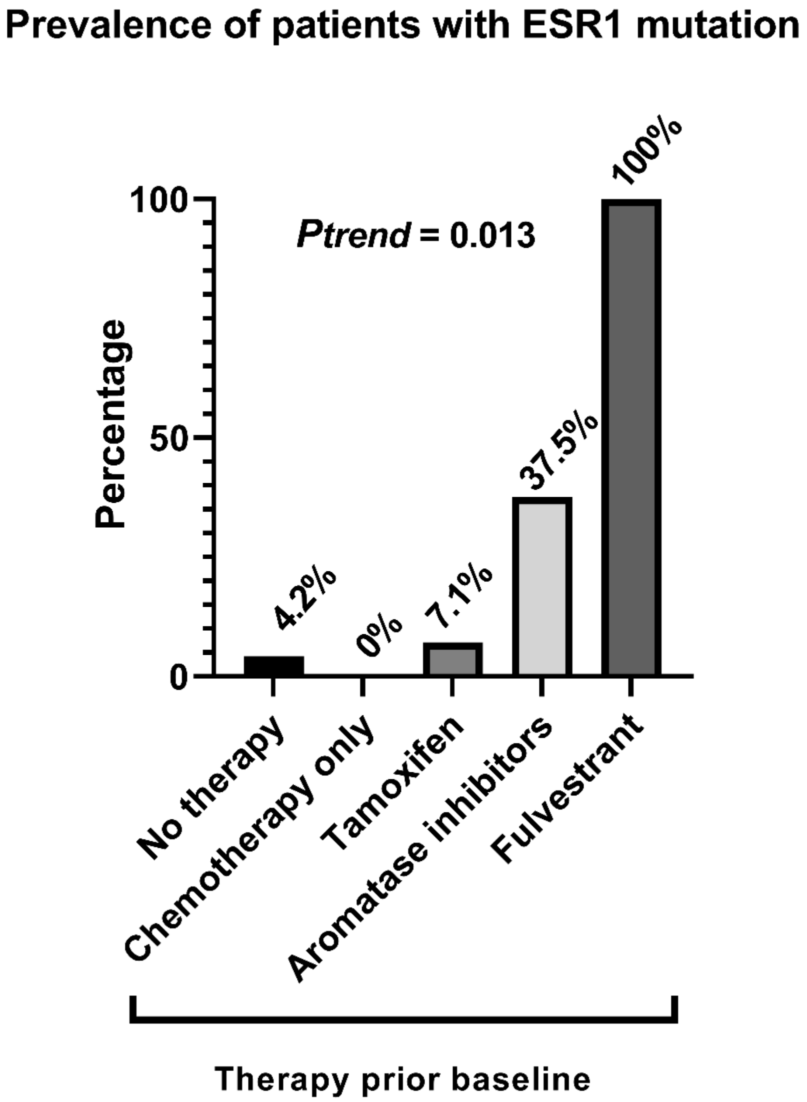

Supplementary Fig. S1: Prevalence of patients with mutated ESR1 with respect to received therapy prior baseline (adjuvant and metastatic setting combined). Overall, 24 patients received no therapy, 3 patients received chemotherapy only, 14 patients received tamoxifen, 16 patients received aromatase inhibitors, and 2 patients received fulvestrant prior baseline sample collection.

Progression free survival

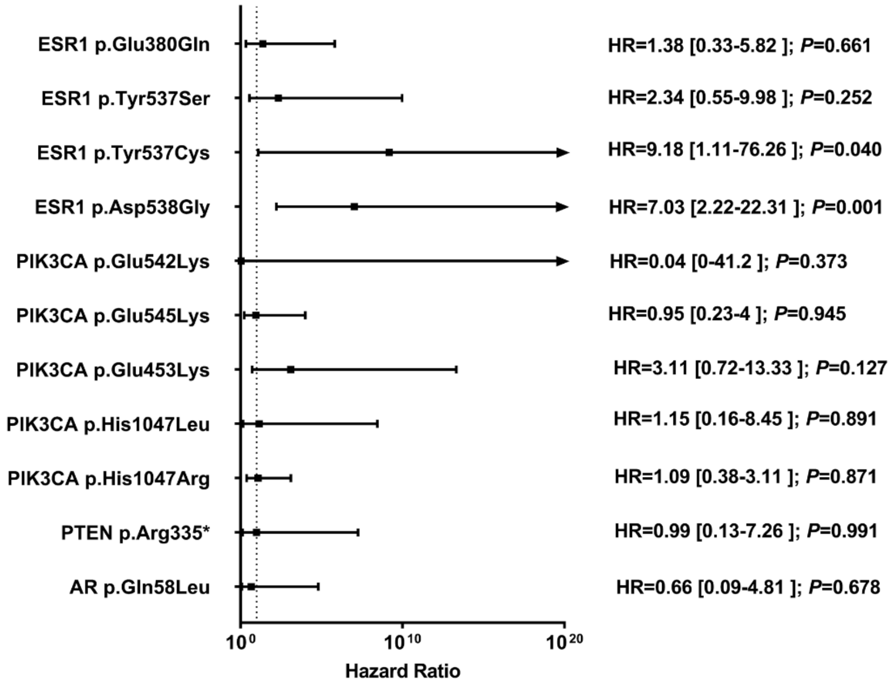

Overall survival

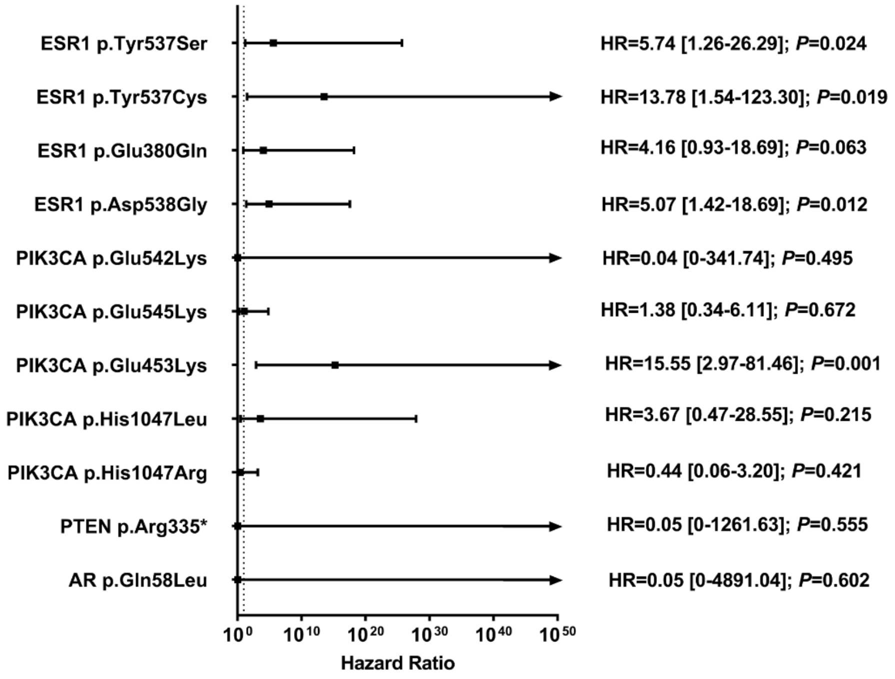

Supplementary Fig. S2: Hazard ratios [95% confidence interval] of individual hotspot mutations for progression free survival and overall survival.

Supplementary Table S1: Characteristics of determined true variants

Please refer to Supplementary Table S1.xlsx

Supplementary Table S2: Genomic targets selected for deep sequencing analysis

Gene	Target region	Chromosomal position (GRCh37 / hg19)
**ESR1**	Exon 7 (hotspot)	chr6:152332817-152332847
	Exon 8 (hotspot)	chr6:152382141-152382195
	Exon 9 (hotspot)	chr6:152415521-152415557
	Exon 10 (complete)	chr6:152419868-152419965
**PIK3CA**	Exon 2 (hotspot)	chr3:178916839-178916980
	Exon 5 (hotspot)	chr3:178921528-178921586
	Exon 8 (complete)	chr3:178927974-178928093
	Exon 10 (complete)	chr3:178935998-178936122
	Exon 21 (complete)	chr3:178951957-178952172
**ERBB2**	Exon 8 (hotspot)	chr17:37868193-37868223
	Exon 19 (complete)	chr17:37880150-37880263
	Exon 20 (complete)	chr17:37880979-37881179
	Exon 21 (complete)	chr17:37881287-37881472
**PTEN**	Exon 1 (coding region)	chr10:89624222-89624311
	Exon 2 (complete)	chr10:89653777-89653872
	Exon 3 (complete)	chr10:89685265-89685320
	Exon 4 (complete)	chr10:89690798-89690852
	Exon 5 (complete)	chr10:89692765-89693014
	Exon 6 (complete)	chr10:89711871-89712024
	Exon 7 (complete)	chr10:89717605-89717783

	Exon 8 (complete)	chr10:89720646-89720881
**TP53**	Exon 2 (coding region)	chr17:7579834-7579918
	Exon 3 (complete)	chr17:7579695-7579727
	Exon 4 (complete)	chr17:7579307-7579596
	Exon 5 (complete)	chr17:7578366-7578560
	Exon 6 (complete)	chr17:7578172-7578295
	Exon 7 (complete)	chr17:7577494-7577614
	Exon 8 (complete)	chr17:7577014-7577161
	Exon 9 (complete)	chr17:7576848-7576932
	Exon 10 (coding region)	chr17:7576620-7576663
	Exon 10 (coding region)	chr17:7576532-7576590
	Exon 11 (complete)	chr17:7573922-7574039
	Exon 12 (coding region)	chr17:7572922-7573014
**KRAS**	Exon 2 (hotspot)	chr12:25398266-25398300
	Exon 3 (hotspot)	chr12:25380275-25380285
**HRAS**	Exon 2 (hotspot)	chr11:534276-534304
	Exon 3 (hotspot)	chr11:533870-533885
**NRAS**	Exon 2 (hotspot)	chr1:115258731-115258758
	Exon 3 (hotspot)	chr1:115256510-115256550
**AR**	Exon 1 (hotspot)	chrX:66765148-66765171
	Exon 1 (hotspot)	chrX:66766123-66766149
